# The safety and efficiency of a 1470 nm laser in obtaining tract hemostasis in tubeless percutaneous nephrolithotomy: a retrospective cross-sectional study

**DOI:** 10.1186/s12894-022-01046-z

**Published:** 2022-07-02

**Authors:** Huihui Zhang, Hanfeng Xu, Kuilin Fei, Dayong Guo, Youjun Duan

**Affiliations:** 1 Department of Urology, Hengyang Medical School, The First Affiliated Hospital, University of South China, 69 Chuanshan Road, Hengyang, 421001 Hunan People’s Republic of China; 2 Institute of Hospital Administration, University of South China, Hengyang, 421001 Hunan People’s Republic of China; 3 Department of Obstetrics, Xiangya Hospital, Central South University, Changsha, 410008 Hunan People’s Republic of China

**Keywords:** Hemostasis, Laser, Tubeless, Percutaneous nephrolithotomy

## Abstract

**Objective:**

It is challenging to perform a tubeless percutaneous nephrolithotomy (PNL) in patients with tract bleeding. The present study was designed to study the safety and efficacy of the 1470 nm laser for hemostatic completion in tubeless PNL patients with tract bleeding.

**Patients and Methods:**

Between January 2020 and October 2021, 120 patients were retrospectively included and divided into two groups. The hemostasis group included 60 patients receiving tubeless PNL, in which a 1470 nm laser was used to manage tract bleeding. The other group included 60 patients receiving tubeless PNL in which the hemostasis procedure was not performed, serving as the control group. The differences in the patients’ demographic characteristics, procedural information, and posttreatment outcomes between the two groups were statistically compared.

**Results:**

The differences associated with sex, age, weight, body mass index, urine culture, stone burden, calyx of puncture, degree of hydronephrosis and comorbidities between the two groups were not statistically significant. Compared with the control group, the hemostasis group showed greatly reduced blood loss (0.61 ± 0.31 vs. 0.85 ± 0.46 g/dL) and decreased postoperative hospitalization duration (2.83 ± 0.81 vs. 4.45 ± 0.91 days). The differences in operative time, stone-free rate, Visual Analogue Score and postoperative complications between the two groups were not statistically significant. In the subgroup analysis, the obese patients and patients with moderate to severe hydronephrosis in the hemostasis group also showed a significantly less blood loss (0.51 ± 0.22 vs. 0.83 ± 0.48 g/dL; 0.54 ± 0.27 vs. 0.85 ± 0.47 g/dL, respectively) and shorter length of postoperative hospitalization (2.62 ± 0.51 vs. 4.47 ± 1.19 days; 2.97 ± 0.63 vs. 4.41 ± 0.91 days, respectively) than those in the control group.

**Conclusions:**

Our results demonstrated that 1470 nm laser is a safe, feasible and effective method to obtain tract hemostasis in tubeless PNL.

## Introduction

Tubeless percutaneous nephrolithotomy (PNL) has been performed to treat kidney and upper ureteral stones since the 1970s and has rapidly evolved as a popular operative approach over time [1]. In many cases, a nephrostomy drainage tube is routinely placed in cases of hemorrhage of the nephrostomy tract and for second-look procedures if necessary. However, PNL can also prolong the length of hospitalization and increase the incidence of postoperative discomfort and postoperative infection [2]. Thus, tubeless PNL has been performed increasingly in recent years. Tubeless PNL is divided into two types: partial tubeless PNL and total tubeless PNL [3]. Partial tubeless PNL is implemented using a ureteral stent without the placement of a nephrostomy drainage tube. Total tubeless PNL is implemented without placement of a nephrostomy drainage tube and a ureteral stent. In most cases, tubeless PNL refers to partial tubeless PNL. For urologists, tract bleeding can be highly worrisome when performing a tubeless PNL. Therefore, if the tract bleeding is eliminated effectively before the end of the operation, a successful tubeless PNL will be guaranteed.

In recent years, lasers have been increasingly used in the field of urology. The 1470 nm diode laser has the superiority of highly efficient tissue vaporization and the creation of a stable coagulation layer [4]. Therefore, the 1470 nm laser vaporization treatment has become an important surgical method to protect patients against BPH [5]. Moreover, the 1470 nm laser appears to be a safe and effective treatment to treat parapelvic renal cysts, due to its excellent hemostatic effects and incision power [6]. In the present study, we aim to present our institutional experience using the 1470 nm laser in tubeless PNL, which appears to be a safe and effective technique to achieve satisfactory hemostasis of tract bleeding.

## Materials and methods

### Patients

A total of 60 patients (35 men and 25 women; 48.0 ± 11.9 years old) admitted to the First Affiliated Hospital of University of South China from January 2020 to October 2021 with renal/upper ureter stones were included in our retrospective cross-sectional study, and were treated with tubeless PNL and hemostasis with the 1470 nm laser. In addition, we selected another 60 matched patients (41 men and 19 women; 45.6 ± 11.0 years old) from the same period with renal/upper ureter stones. As the control group, they received traditional tubeless PNL but without hemostasis with the 1470 nm laser. The inclusion criteria were as follows: the presence of renal or upper ureter stones (diameter < 3.5 cm) based on imaging examination; no history of surgery in the ipsilateral urinary tract; lack of ureteral stricture; and no uncontrolled infection. The exclusion criteria were as follows: younger then 18 years old; requiring a second-look PNL; patients with severe heart or renal failure or diabetes; with spinal deformity; or with conditions that could lead to bleeding, such as aspirin consumption, nonsteroidal anti-inflammatory drug consumption, and coagulation dysfunction.

Preoperative radiological investigations were evaluated via ultrasonography, X-ray of the kidney, ureter, and bladder (KUB) region and computed tomography (CT) scan. Routine blood tests, liver function, renal function, coagulation function, blood glucose, urinalysis and urine culture were performed preoperatively. Appropriate antibiotics, such as quinolones or cephalosporins, were administered preoperatively according to positive urinalysis and urine culture results. The assessment of hydronephrosis was based on the Society of Fetal Urology grading system [7]. The stone burden was calculated by the formula (0.785 × length_max_ × width_max_) introduced by the Clinical Research Office of the Endourological Society [8]. According to the standard set by the World Health Organization, a body mass index (BMI) of ≥ 28 is considered an obese patient in the Chinese population [9]. The present study was approved by the Ethics Committee of the First Affiliated Hospital of University of South China (No.202008191), in accordance with the Declaration of Helsinki. Written informed consent to perform the surgery and to publish the clinical data was obtained from the patients.

### Surgical procedure

First, the patient was placed in the lithotomy position under general anesthesia. A 4F ureteral catheter was inserted into the renal pelvis of the surgical side by a 8/9.8F semirigid ureteroscope (Karl Storz, Germany). Second, the patient was turned to the prone position. Puncture was performed with an 18-G needle under the guidance of sonography (Hitachi, Japan). Access to the targeted calix was confirmed by aspirating the urine, and then a safe guidewire (Kang Yi Bo, China) was inserted. The tract was dilated with serial fascial dilators from 8 to 18F, and an 18F peel-away sheath was smoothly placed in the tract. A 8/9.8F semirigid ureteroscope was used to find the stones. Lithotripsy was performed using a holmium laser with a 600-μm fiber at an energy level of 50 W and a frequency level of 20 Hz. Stone fragments were eliminated with saline perfusion.

**Fig. 1:**
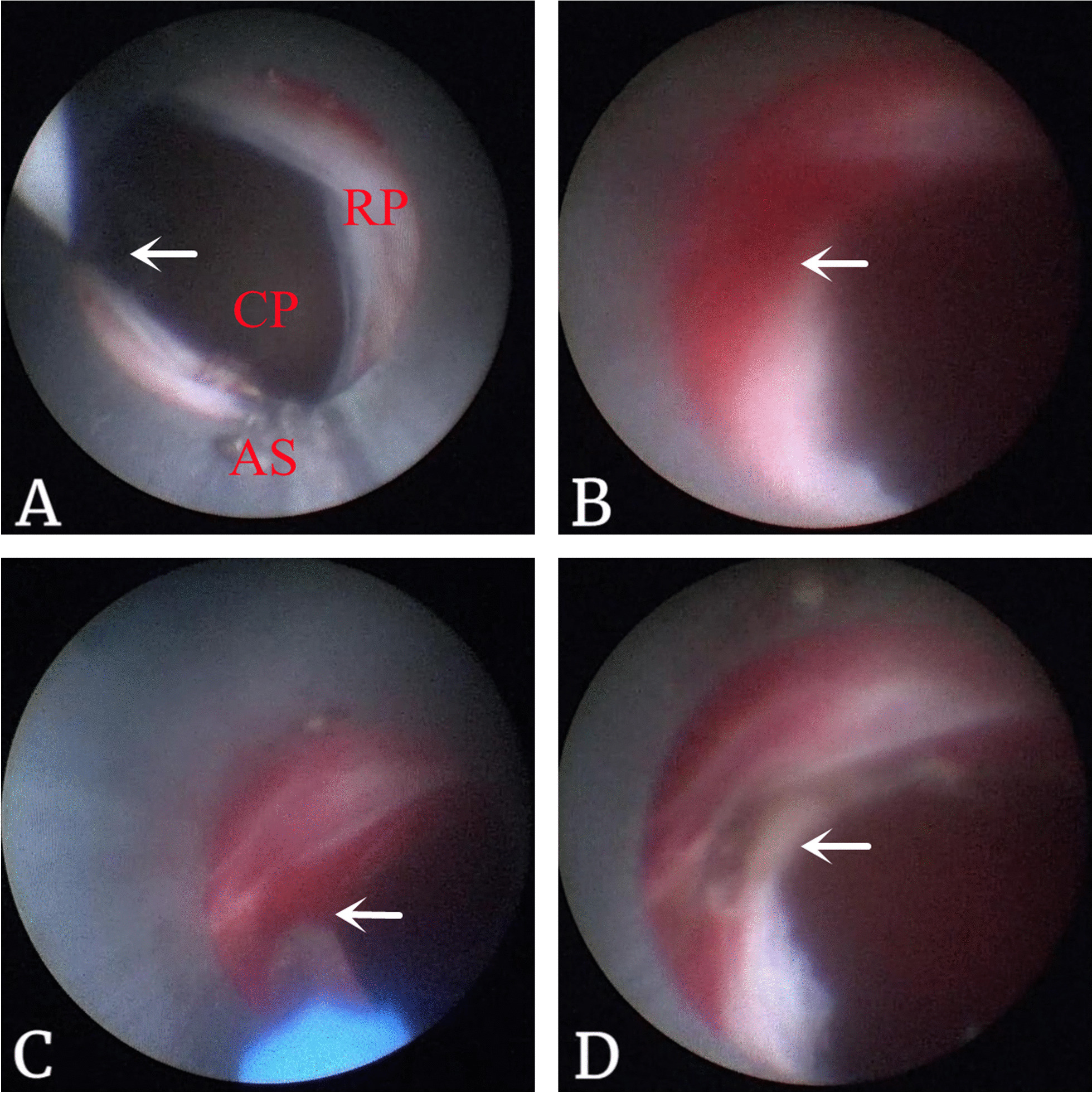
The hemostasis management of tract bleeding with 1470 nm laser in tubeless PNL. **A** The safe guidewire (the arrow) was inserted into the calyx of puncture (CP), then the access sheath (AS) was drawn back slowly to find the bleeding spot of the renal parenchyma (RP). **B** The arrow showed a bleeding spot. **C** The 1470 nm laser fiber (the arrow) was introduced to eliminate the bleeding spot. **D** The surface of the bleeding spot was clear after the hemostasis management (the arrow)

After fluoroscopic confirmation of stone clearance, a double-J stent was placed. In the control group, the sheath was then removed immediately, and a dressing was placed on the percutaneous access site. However, in the hemostasis group, before removing the sheath, the safe guidewire was inserted into the tract again (Fig. 1A). Then, we withdrew the sheath slowly to detect suspicious bleeding spots. The renal parenchyma, perinephric fat and muscular layers were checked carefully in sequence. If a bleeding spot was found (Fig. 1B), the 1470 nm laser fiber was introduced to eliminate the bleeders (Fig. 1C, D), at a coagulation energy level of 50 W. The manufacturer of the 1470 nm laser machine is Wuhan Miracle Laser Technology Co., Ltd from China.

### Follow-up

The clinical data and the surgical outcomes were collected and recorded. We then analyzed factors such as sex, age, weight, BMI, side of stone, urine culture, stone burden, calyx of puncture, grade of hydronephrosis and comorbidities. In addition, the conditions of postoperative factors, such as the Visual Analogue Score (VAS), operation time, blood loss, stone-free rate, hospital stay, fever, peritoneum injury, blood transfusion, and delayed bleeding, were collected. The operative time was documented as the time from the first puncture until the final placement of the dressing. Residual stones were assessed postoperatively by KUB and CT scans on day one or day two in all patients. The stone-free rate (SFR) was defined as residual stone fragments less than 4 mm in diameter. The mean hemoglobin drop was calculated according to the formula used by Stoller [10]. All patients visited our department at 1 month postoperatively. If delayed bleeding did not occur, the double-J stent was removed.

### Statistical analysis

SPSS statistical software, version 22.0, was used for the analysis (SPSS Inc., Chicago, IL, USA). The mean ± standard deviation was used to describe the measurement data. Student's t test or the Mann–Whitney U test was used to analyze the numerical variables between the two groups. Categorical variables were assessed by the two-tailed chi-squared test or Fisher's exact test, as appropriate. P < 0.05 was considered statistically significant.

## Results

A total of 120 patients were included in this study (60 patients in each group). The detailed characteristics and clinical data are described in Table 1. The patients’ baseline features in both the hemostasis and control groups were not significantly different in any aspects,such as sex, age, weight, BMI, urine culture, stone burden, calyx of puncture, degree of hydronephrosis and comorbidities.

**Table 1:** The basic characteristics of the patients

Parameters	The control group (n = 60)	The hemostasis group (n = 60)	P-value
Sex (n, %)			0.256
Male	41(68.3%)	35(58.3%)	
Female	19(31.7%)	25(41.7%)	
Age (years,mean ± SD)	45.6 ± 11.0	48.0 ± 11.9	0.261
Weight (kg,mean ± SD)	66.2 ± 6.8	65.4 ± 6.0	0.461
BMI (kg/m^2^,mean ± SD)	25.2 ± 3.0	25.7 ± 2.9	0.363
Side (n, %)			0.461
Right	28(46.7%)	24(40.0%)	
Left	32(53.3%)	36(60.0%)	
Urine culture (n, %)			0.591
Positive	9(15.0%)	7(11.7%)	
Negative	51(85.0%)	53(88.3%)	
Stone burden (mm^2^, mean ± SD)	611.4 ± 282.4	562.8 ± 274.0	0.341
Calyx of puncture			0.442
Upper calyx	34(56.7%)	27(45.0%)	
Middle calyx	22(36.7%)	28(46.7%)	
Lower calyx	4(6.7%)	5(8.3%)	
Grade of hydronephrosis			0.584
Mild to Moderate	28(46.7%)	31(51.7%)	
Moderate to severe	32(53.3%)	29(48.3%)	
Comorbidities			0.764
Hypertension	4(6.7%)	4(6.7%)	
Diabetes mellitus	5(8.3%)	3(5.0%)	

The surgical characteristics and follow-up outcomes are summarized in Table 2. The operative time was recorded as the time from the initial ultrasound-guided puncture to the placement of the incision dressing. The average operative time in the hemostasis group was 43.4 ± 14.0 min, and it was 41.9 ± 17.0 min in the control group. Although the operation time in the hemostasis group seemed to be slightly longer, the difference was not significant. The stone-free rate in the hemostasis group (53/60, 88.3%) was similar to that in the control group (56/60, 93.3%). Notably, compared with the control group, the hemostasis group showed markedly reduced blood loss (0.61 ± 0.31 g/dL vs. 0.85 ± 0.46 g/dL, P = 0.001) and decreased postoperative length of hospitalization (2.83 ± 0.81 days vs. 4.45 ± 0.91 days, P = 0.001). Blood transfusion was not observed in the two groups. Moreover, the conditions of VAS and postoperative complications were comparable between the groups (P = 0.781 and P = 0.433, respectively). The control group experienced 2 cases of fever and 1 case of peritoneum injury, while the hemostasis group experienced 4 cases of fever. All of these cases were cured by conservative treatment only. At 1 month postoperatively, no delayed bleeding occurred in the two groups.

**Table 2:** The surgical characteristics and outcomes of the control and hemostasis group

Surgical characteristics and outcomes	The control group (n = 60)	The hemostasis group (n = 60)	P-value
Operative time (min, mean ± SD)	41.9 ± 17.0	43.4 ± 14.0	0.598
Mean estimated blood loss (g/dL, mean ± SD)	0.85 ± 0.46	0.61 ± 0.31	0.001
Stone-free rate (n, %)	53(88.3%)	56(93.3%)	0.343
Postoperative hospitalization time (day, mean ± SD)	4.45 ± 0.91	2.83 ± 0.81	0.001
VAS (mean ± SD)	3.17 ± 0.74	3.03 ± 0.76	0.781
Postoperative complication (n, %)			0.433
Fever	2(3.3%)	4(6.7%)	
Peritoneum injury	1(1.7%)	0	
Blood transfusion	0	0	
Delayed bleeding 1 months postoperatively	0	0	

The effects of employing the 1470 nm laser on reducing the amount of blood loss and the length of postoperative hospitalization were also pronounced in obese patients and those with moderate to severe hydronephrosis (Table 3). In the obese patients, compared with the control group, the hemostasis group showed markedly reduced blood loss (0.51 ± 0.22 g/dL vs. 0.83 ± 0.48 g/dL, P = 0.022) and decreased length of postoperative hospitalization (2.62 ± 0.51 days vs. 4.47 ± 1.19 days, P = 0.013). In patients with moderate to severe hydronephrosis, compared with the control group, the hemostasis group also showed markedly reduced blood loss (0.54 ± 0.27 g/dL vs. 0.85 ± 0.47 g/dL, P = 0.003) and decreased length of postoperative hospitalization (2.97 ± 0.63 days vs. 4.41 ± 0.91 days, P = 0.008).

**Table 3:** The surgical characteristics and outcomes of patients with obesity or hydronephrosis

Surgical characteristics and outcomes	The control group	The hemostasis group	P-value
Obese patients	n = 15	n = 13	
Mean estimated blood loss (g/dL, mean ± SD)	0.83 ± 0.48	0.51 ± 0.22	0.022
Postoperative hospitalization time (day, mean ± SD)	4.47 ± 1.19	2.62 ± 0.51	0.013
Moderate to severe hydronephrosis patients	n = 32	n = 29	
Mean estimated blood loss (g/dL, mean ± SD)	0.85 ± 0.47	0.54 ± 0.27	0.003
Postoperative hospitalization time (day, mean ± SD)	4.41 ± 0.91	2.97 ± 0.63	0.008

## Discussion

Currently, although tubeless PNL has attracted increasing attention, it should be conducted in strictly selected patients with no complex renal stones, no abnormal coagulation function, no urinary tract stenosis, no noticeable hemorrhage during the operation, and no major collecting-system injury [11]. For urologists, there are unavoidable concerns that potential postoperative bleeding in the working channel without compression of a nephrostomy tube could occur. Therefore, it is important to reduce the intraoperative bleeding for the safety of tubeless PNL. In the present study, we demonstrated a novel and effective method to obtain tract hemostasis in tubeless PNL using the 1470 nm laser, with less blood loss and a shorter hospital stay.

Improvements in surgical technique and instrumentation have contributed greatly to decreasing the bleeding in PCNL. The access sheath for PNL has been miniaturized in recent years. According to the size of the access sheath, PNL is categorized into standard PCNL(24-30F), mini-PCNL (≤ 22F), Chinese mini-PCNL(14-20F), super-mini-PCNL(10-14F), and so on [12]. Several studies have reported that mini-PCNL has overriding advantages of less bleeding and postoperative pain, similar SFR, and lower complication rates compared with the standard PCNL [13, 14]. Thus, the F18 access sheath was used in the present study, achieving a high stone-free rate and mild complications. Although a systematic review revealed no significant difference between ultrasonography and fluoroscopy regarding bleeding [15], we prefer ultrasonography-guided PNL, because the advantages of using the ultrasonography for guidance include decreased exposure to radiation and lower overall cost, decreased operation time and detection of radiolucent stones [16, 17]. Moreover, it is useful for decreasing the rate of visceral injury due to better visualization of the surrounding viscera and the depth of needle penetration, and for avoiding vascular injury by adding Doppler flow imaging [18]. In the present study, we conducted ultrasonography-guided PNL for all the patients. None of them experienced obvious intraoperative bleeding or required blood transfusions.

In recent years, some novel minimally invasive technologies have been used recently to prevent postoperative bleeding in the absence of an indwelling nephrostomy tube. Shah [19] instilled a fibrin sealant and gelatin matrix hemostatic sealant. Kumar [20] used a ‘Santosh-PGI hemostatic seal’ to seal off the access tract after the procedure of PNL, which decreased bleeding and urinary extravasation. Electrocauterization with a roller barrel electrode and a clear amplatz renal sheath was also reported to be a method to obtain hemostasis after completion of a tubeless PNL [21]. However, because the resources of hemostatic sealants are limited and the use of a roller barrel electrode is not convenient, the methods above are far from perfect. The 1470 nm laser previously demonstrated faster sealing and cutting of blood vessels with lower thermal spread than holmium and other laser types [22]. It works at a wavelength of 1470 nm which is absorbed by both water and hemoglobin. Due to its excellent tissue vaporization capacity and great coagulation properties, the 1470 nm laser has been widely used in the operative management of benign prostatic hyperplasia, with demonstrated safety and efficacy [5]. Similarly,our results showed that the patients treated with the 1470 nm laser for hemostasis had a smaller decrease in hemoglobin and shorter hospital stay than patients undergoing routine tubeless PNL. Compared with electrocautery as described before, we used saline for dripping irrigation instead of mannitol. The device is simpler and requires less time. Although a previous study demonstrated the feasibility of sealing renal vessels in an in vivo porcine model using 1470-nm laser [23], to the best of our knowledge, the current study was the first to explore the feasibility of using 1470 nm laser to eliminate bleeding in the tract during tubeless PNL.

Severe hydronephrosis will cause thinning of the renal parenchymal over time and eventually lead to perinephritis [24]. Then, severe bleeding after PNL can be caused by infection. Moreover, when the renal cortical thickness is less than 4 mm, bleeding is very common in these patients because the thin renal cortex has difficulty shrinking and healing [25]. Encouragingly, our study found that all patients in the hemostasis group had mild hematuria, even for patients with moderate to severe hydronephrosis. Contradictory results from Kim’s study demonstrated that the absence of hydronephrosis was a significant risk factor for blood transfusion when performing PNL [26]. Similarly, in another study, no relationship was detected between renal parenchymal thickness and bleeding [27]. The possible explanations for the contradiction could be the different selections of calyx, puncture and tract dilatation, since they also affect bleeding. For example, a randomized clinical trial indicated that the one-stage tract dilation technique might cause more parenchymal damage than the gradual dilation technique [28].

Another one of the main concerns regarding blood loss during PNL is BMI. Lee et al. [29] demonstrated that high BMI was significantly associated with the risk of severe bleeding during PNL. It is also not surprising that obesity can complicate the puncture and tract dilation, and therefore lead to repeated manipulations during PNL which could be more traumatic to the renal vascular system and parenchyma. Encouragingly, our study found that all patients had mild hematuria, even patients with BMI ≥ 28. None of the patients required a transfusion or experienced severe perioperative complications. During 1 month of follow-up, delayed bleeding did not occur. Therefore, our hemostasis technique could enable us to perform tubeless PNL more safely.

Despite all of its minimal invasiveness and good results, the difficulties of our hemostasis procedure are also obvious. The poor vision and operating space in the surgery are the most common inconveniences for surgeons [30]. In our experience, we controlled the water pressure by saline dripping to keep the vision clear. The sheath was drawn back slowly to find the bleeding spots. The renal parenchyma, perinephric fat and muscular layers were checked carefully. Then, a 1470 nm laser was introduced to eliminate the bleeders. This step could explain why the operation time in the hemostasis group was slightly longer than that in the control group. Another problem with the procedure is the regulation of power. It will be very difficult to avoid bleeding using a low-power laser. The 1470 nm laser is easily absorbed by hemoglobin and water, allowing heat to be concentrated in a small piece of tissue with a penetration depth of 2–3 mm [31]. In our study, the 1470 nm laser generator had settings of 50 W for coagulation in most cases. We could raise the power when necessary. This high-power characteristic significantly reduced intraoperative bleeding.

In the present study, the procedure using 1470 nm laser to obtain tract hemostasis in tubeless PNL was advantageous in that it provided markedly reduced blood loss and a shorter hospital stay. However, we must admit that this study carries a major limitation in that it was a retrospective, cross-sectional, small samplesize study performed at a single center. In addition, the pooled results relative to hospital stay could have resulted in a potential bias because they could be easily impacted by the patients’ individual thoughts and requirements. Moreover, although all surgeons had more than 10 years of experience with PNL, the surgeon’s skills might also have an influence on the operative time. Considering the limitations mentioned above, a prospective, randomized study with a larger sample size will be more conclusive.

In conclusion, in terms of good effects and mild complications (Clavien-Dindo grade I or II), the current study demonstrated that the 1470 nm laser is a novel, safe, and efficient technology for hemostasis of the nephrostomy tract in tubeless PNL.

## Data Availability

The datasets used and analysed in the current study are from the corresponding author on reasonable request.
